# Supplementary material for: Polyketide synthase gene clusters in the ericoid mycorrhizal fungus Oidiodendron maius: genome-wide prediction, expression profiling, and functional characterization of knockout mutants
Source: Front Plant Sci. 2026 Sep 16;17:1924430. doi: 10.3389/fpls.2026.1924430 (PMC13623903; doi:10.3389/fpls.2026.1924430)
Supplement: Supplementary file 1 [file SupplementaryFile1.zip › Supplementary Materials/Supplementary Folder 1.zip!/Oidma1_AssemblyScaffolds_Repeatmasked/knownclusterblast/cluster14/jgi.p_Oidma1_40064_mibig_hits.html]

| MIBiG Protein | Description | MIBiG Cluster | MiBiG Product | % ID | % Coverage | BLAST Score | E-value |
| --- | --- | --- | --- | --- | --- | --- | --- |
| ACB12553.1 | Fum6 | BGC0000063 | Fumonisin\_biosynthetic\_gene\_cluster | 27 | 40.3041825095 | 67 | 6.7e-11 |
| AAG27132.1 | Fum6p | BGC0000062 | Fumonisin\_biosynthetic\_gene\_cluster | 28 | 40.4942965779 | 62 | 1.6e-09 |
| AIA58897.1 | putative\_cytochrome\_P450 | BGC0001141 | Brefeldin\_biosynthetic\_gene\_cluster | 22 | 46.5779467681 | 55 | 2e-07 |
